# Next generation mouse models of squamous cell lung cancer for translational immuno-oncology

**DOI:** 10.18632/oncotarget.27828

**Published:** 2020-12-01

**Authors:** Josephine Hai

**Keywords:** squamous cell lung cancer, mouse models, immunotherapy, CRISPR

With immunotherapy rapidly becoming a mainstay of cancer treatment, a more comprehensive understanding of the immune microenvironment is essential. Patient-derived xenografts (PDX) lack the endogenous immune microenvironment, and humanized mice, though promising, often do not accurately recapitulate basic immunological features, such as correct abundance of major leukocytic lineages. Genetically engineered mouse models (GEMMs) and transplantable tumors to syngeneic immunocompetent mice are often necessary to test novel immunotherapies and identify potential predictive biomarkers.

Historically, the development of therapies for lung squamous cell carcinoma (LSCC) has significantly lagged, due in part to the lack of animal models with pure squamous histology and patient-relevant mutant genotypes. The development of LSCC GEMMs has been met with many challenges. For one, *in vivo* interrogation of the complex mutational landscape of LSCC, which was only revealed in 2012 from The Cancer Genome Atlas (TCGA), has been limited by the resource-intensive need to generate and crossbreed mice bearing multiple, distinct engineered inducible mutant alleles [1]. Unlike lung adenocarcinoma, no single driver oncogene has been validated for LSCC. Furthermore, these GEMMs often require tissue-specific *cre*-lox systems, which pose challenges for squamous cell carcinomas since this can lead to premature termination of mice that develop skin lesions before the onset of LSCCs [2]. Xu *et al*. previously demonstrated that bi-allelic inactivation of *Pten* and *Stk11* in mice gave rise to LSCCs with high PD-L1 expression after 40 to 50 weeks [3]. Ferone *et al*. have also developed LSCC transgenic mice harboring *Sox2* overexpression with bi-allelic inactivation of *Pten* and *Cdkn2ab* after 7–9 months with a 73% penetrance [4]. The long latency periods for the development of LSCC in GEMMs (7–9 months) significantly hampers preclinical testing of immunotherapies. Additionally, cell lines derived from the primary murine tumors do not always grow in culture to enable transplantable syngeneic tumor models, presumably due in part to p53-activation dependent growth arrest, and require subsequent steps of artificial immortalization with SV40 T-antigen [5].

To overcome these barriers, we and others have efficiently engineered genetically-defined immunocompetent mouse models by leveraging both organoid and CRISPR-based methodology [6–8]. We engineered lung organoids derived from *Cre*-dependent *Sox2* knock-in mice bearing three tumor suppressor gene mutations co-occurring in human LSCC (*Tp53*^indel^/*Pten*^indel^/*Cdkn2a*^indel^) and capable of forming tumors in syngeneic immunocompetent hosts with significantly shorter latencies than traditional crossbreeding strategies [6]. These transformed organoids give rise to SCC that closely mirror human tumor histology, signaling alterations, and immune-stromal-tumor microenvironment [6]. Using this new immunocompetent mouse model, we demonstrated efficacy of combining immune checkpoint inhibitors with DNA-damage inducing targeted therapy for this genotype of LSCC [6].

Rapid generation of a panel of immunocompetent mouse models of complex genotypes using this approach would be a powerful platform for characterizing genotype-specific sensitivities to immunotherapies for LSCC and could be adapted to high throughput drug screens. Studies from other cancer types have begun to bring this concept to fruition. O’Rourke *et al*. previously highlighted that use of *ex vivo* engineered mouse organoids derived from GEMMs provided a flexible, fast, and low-cost platform to model all stages of colorectal cancer that recapitulated the entire adenoma-adenocarcinoma-metastasis axis *in vivo* [8]. More recently, Zhang *et al*. generated multiple high grade serous tubo-ovarian carcinoma (HGSC) models exhibiting a panel of genotypes frequently observed in patients by CRISPR engineering fallopian tube epithelial (FTE) organoids derived from *Tp53^f/f^* GEMMs [7]. Using these syngeneic organoid models, they demonstrated that tumors with *Tp53^–/–^*/*Ccne1^OE^*/*Akt2^OE^*/*Kras^OE^* genotype were highly sensitive to chemotherapy and immunotherapy combinations (gemcitabine plus anti-PD-L1 and anti-CTLA-4), while *Tp53^–/–^*/*Pten^–/–^*/*Nf1^–/–^* tumors were not [7]. Similarly, the Weinberg group also demonstrated the utility of engineering *ex vivo* murine FTE cells bearing complex mutational combinations (up to five co-occurring genetic alterations) and forming a panel of five genotype-specific models that grew in syngeneic hosts [9]. By treating these new mouse models with a combination of immune checkpoint inhibitors and chemotherapies, they identified follistatin as a driver of resistance to immune checkpoint inhibitors in *Ccne1*-overexpressing (*Ccne1^OE^*) mice [9].

Leveraging emerging technologies in both CRISPR/Cas9 mutagenesis and organoid culturing methods offers hope that enhanced development of unique clinically relevant mouse models will shed light on the complex tumor-immune microenvironment, enabling more efficient preclinical testing to understand how different patient subsets may respond to novel immunotherapies. This powerful approach holds promise to predict genotype-driven drug responses in a personalized fashion, especially for LSCC patients, who have had very limited therapeutic options for many years.
